# Supplementary material for: Spontaneous voltage and persistent electric current from rectification of electronic noise in cuprate/manganite heterostructures
Source: Nat Commun. 2025 Jul 1;16:5900. doi: 10.1038/s41467-025-61014-7 (PMC12215835; doi:10.1038/s41467-025-61014-7)
Supplement: Supplementary file 1 — Supplementary Information [file 41467_2025_61014_MOESM1_ESM.pdf]

Supplementary Information

for

Spontaneous voltage and persistent electric current from rectification of electronic noise in cuprate/manganite heterostructures

Mathias Soulier[1], Shamashis Sengupta[2], Yurii G. Pashkevich[1,3], Roxana Capu[4], Ryan Thompson[1], Jarji Khmaladze[1], Miguel Monteverde[5], Louis Dumoulin[2], Dominik Munzar[6], Christian Bernhard[1*], Subhrangsu Sarkar[1*]

[1] University of Fribourg, Department of Physics and Fribourg Center for Nanomaterials, Chemin du Musée 3, CH-1700 Fribourg, Switzerland
[2] Université Paris-Saclay, CNRS/IN2P3, IJCLab, 91405 Orsay, France
[3] O. Galkin Donetsk Institute for Physics and Engineering NAS of Ukraine, 03028 Kyiv, Ukraine
[4] West University of Timisoara, Faculty of Physics, Bd Vasile Parvan 4, Timisoara-300223, Romania
[5] Université Paris-Saclay, CNRS, Laboratoire de Physique des Solides, 91405, Orsay, France
[6] Department of Condensed Matter Physics, Faculty of Science, Masaryk University, Kotlářská 2, 61137 Brno, Czech Republic

*Corresponding author
Christian Bernhard (christian.bernhard@unifr.ch), Subhrangsu Sarkar (subhrangsu.sarkar@unifr.ch)

Supplementary Notes

1. Phase Diagram of $Nd_{1-x}(Ca_{1-y}Sr_y)MnO_3$ 3
2. Seeback Voltage 4
3. Dependence of Spontaneous Voltage on the driving mode of the PPMS magnet 5
4. Evidence of rectification of external noise 6
5. Temperature and magnetic field dependence of the second harmonic signal 7
6. Electrical characterization of a 10 nm thick NCSMO layer 8
7. Estimate of the depth of the rachet potential 8

Supplementary Note 1: Phase Diagram of $Nd_{1-x}(Ca_{1-y}Sr_y)MnO_3$

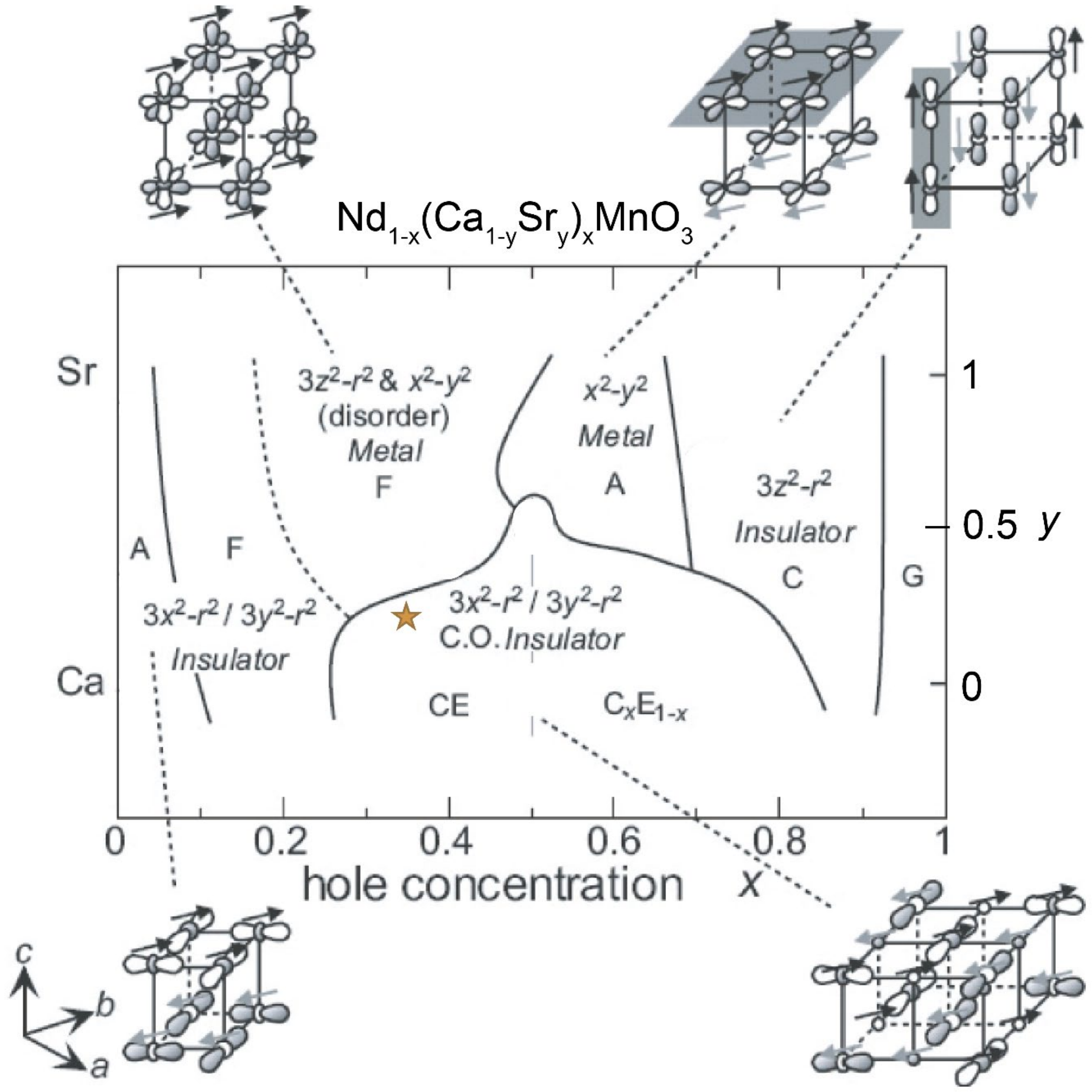

*Supplementary Figure 1:**Phase diagram of $Nd_{1-x}(Ca_{1-y}Sr_y)MnO_3$**: The yellow star shows how the NSCMO layers of our heterostructures are positioned in the phase diagram of rare-earth perovskite manganites[1] (Used with permission of [IOP Publishing, Ltd], from [Critical features of colossal magnetoresistive manganites, Y Tokura, volume 69, edition no. 3 (2006)]; permission conveyed through Copyright Clearance Center, Inc.).*

Supplementary Note 2: Seeback Voltage

When a sample is cooled down in contact to a thermal bath with metal wires attached to it, there can be a substantial temperature gradient between the contacts – especially if the cooling rate is large. This effect is typically larger at higher temperature and gets smaller towards low temperature. This thermal gradient can produce a measurable voltage due to the so-called Seeback-effect. The voltage signal due to this Seeback-effect disappears as the temperature of the sample is stabilized and the temperature gradient thus vanishes.

In order to estimate the maximum contribution of this Seeback effect, we cooled down a standard thin-film resistor of 1 kΩ from 320 K to 10 K in 3 steps (320-200 K, 200-100 K and 100-10 K) at a rate of 7 K/min (maximum possible cooling rate of our PPMS). At the end of each step, we waited for 300s at the same temperature and recorded the Seeback voltage as a function of time.

As shown in Supplementary Fig. 2, we observe a Seeback voltage, V^{SB}, with an overall magnitude on the order of a few microvolts that decreases in magnitude toward low temperature. Moreover, the value of V^{SB} is strongly reduced as the temperature is stabilized at the end of each step. Note that the maximal value of V^{SB} of about 9 μV is about three orders of magnitude smaller than the spontaneous voltage of the N10Y7-SL reported in our manuscript.

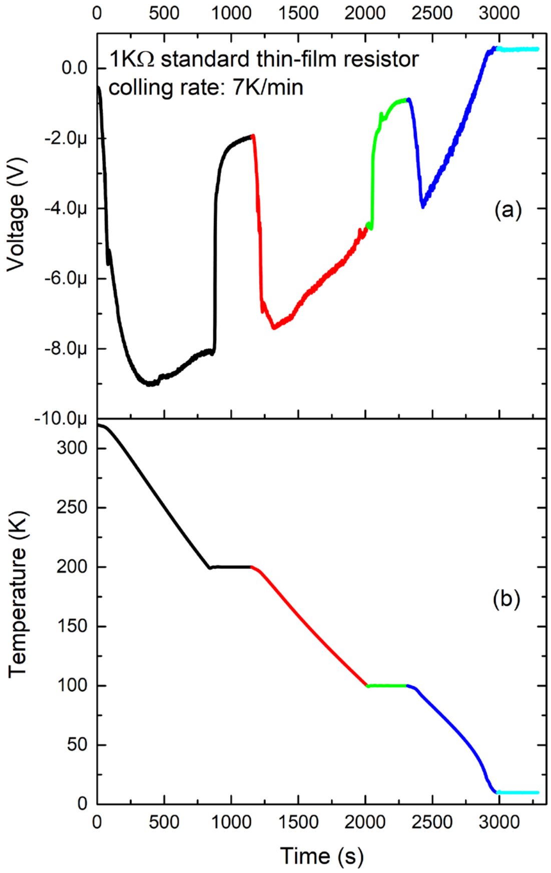

Supplementary Figure 2: ***Seeback effect of standard thin-film resistor of 1 KΩ during cooling in a PPMS at a maximal rate of 7 K/min****. Panel (a) shows the thermoelectric voltage and (b) the reading of the temperature sensor of the PPMS. Black, red and blue lines represent the 3 cooling steps from 320-200 K, 200-100 K and 100-10 K respectively, whereas the intermediate black, green and cyan regions denote stabilization periods when the cooling was stopped.*

Supplementary Note 3: Dependence of Spontaneous Voltage on the driving mode of the PPMS magnet

To confirm the hypothesis that the SpV signal arises from the rectification of electronic noise we verified that SpV varies when we change the driving mode of the PPMS magnet which is known to alter the electronic noise level. The NYN-SL was first cooled to 60 K without any applied voltage/current. We then first recorded SpV for a duration of 3600 seconds with the magnet set to zero in persistent mode in order to determine the time-averaged value at the lowest

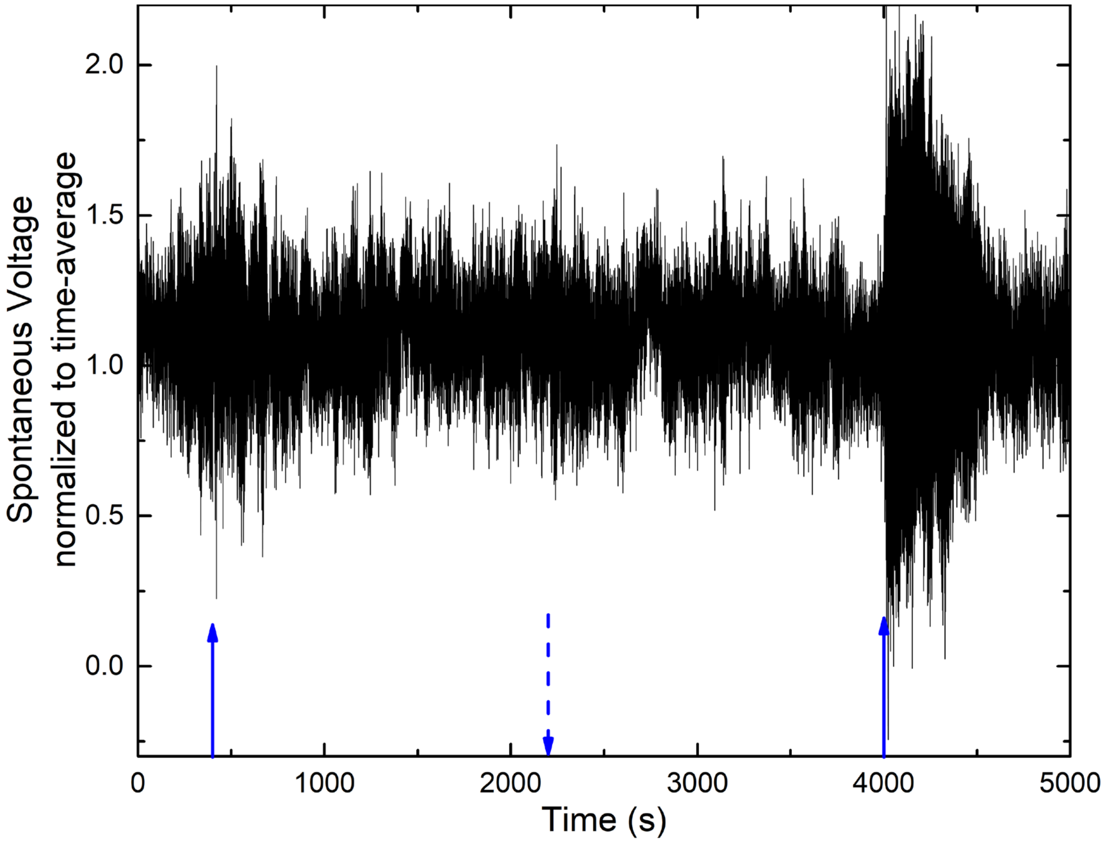

Supplementary Figure 3: ***Variation of the SpV signal upon changing the driving mode of the PPMS magnet and the subsequent electronic noise level****. The solid blue upward arrow marks the time at which the magnetic was switched from persistent mode with a lower noise level to driven mode with a higher noise level. The dashed blue downward arrow marks the inverse change of the driving mode.*

noise level. The latter value was used to normalize the subsequent measurement of the time evolution of SpV while the operational mode of the magnet power supply was switched from persistent mode (low noise level) to driven mode (higher noise level), as shown in Supplementary Figure 3 by the solid and dashed blue arrows, respectively.

In particular, the solid up arrow marks the time at which the magnet was set to driven mode at 12 Oe and the dashed down arrow when it was set back to persistent mode at zero field. It is evident from these data that SpV (and its fluctuations) is enhanced when the magnet is operated in driven mode which gives rise to a higher noise level.

Supplementary Note 4: Evidence of Rectification of External Noise

A noise inducing coil has been placed above the sample as shown as in Supplementary Figure 4(a). It was used to expose the sample to a broadband (50MHz) white noise that was transmitted through this coil set from an ac function generator, while the sample was cooled down to different measurement temperatures. Two probes of a Keithley digital multimeter were connected to the two-line contacts on the sample (in the same geometry as the measurement of SpV). Supplementary Figure 4(b) shows that there is a large difference in the SpV between the 'ON' and 'OFF' states of the function generator. Further, this difference increases while reducing sample temperature down to 30K, albeit showing a reduction around 80K. Measurement of this pick-up signal as a function of the frequency of a sine-wave drive signal through the noise inducing coil indicates a broadband rectification of signals for ~3-12MHz bandwidth. However, at T<80K, we also see an overall increase in the background of this frequency spectrum indicating a pick-up from a different origin – most likely at higher frequencies.

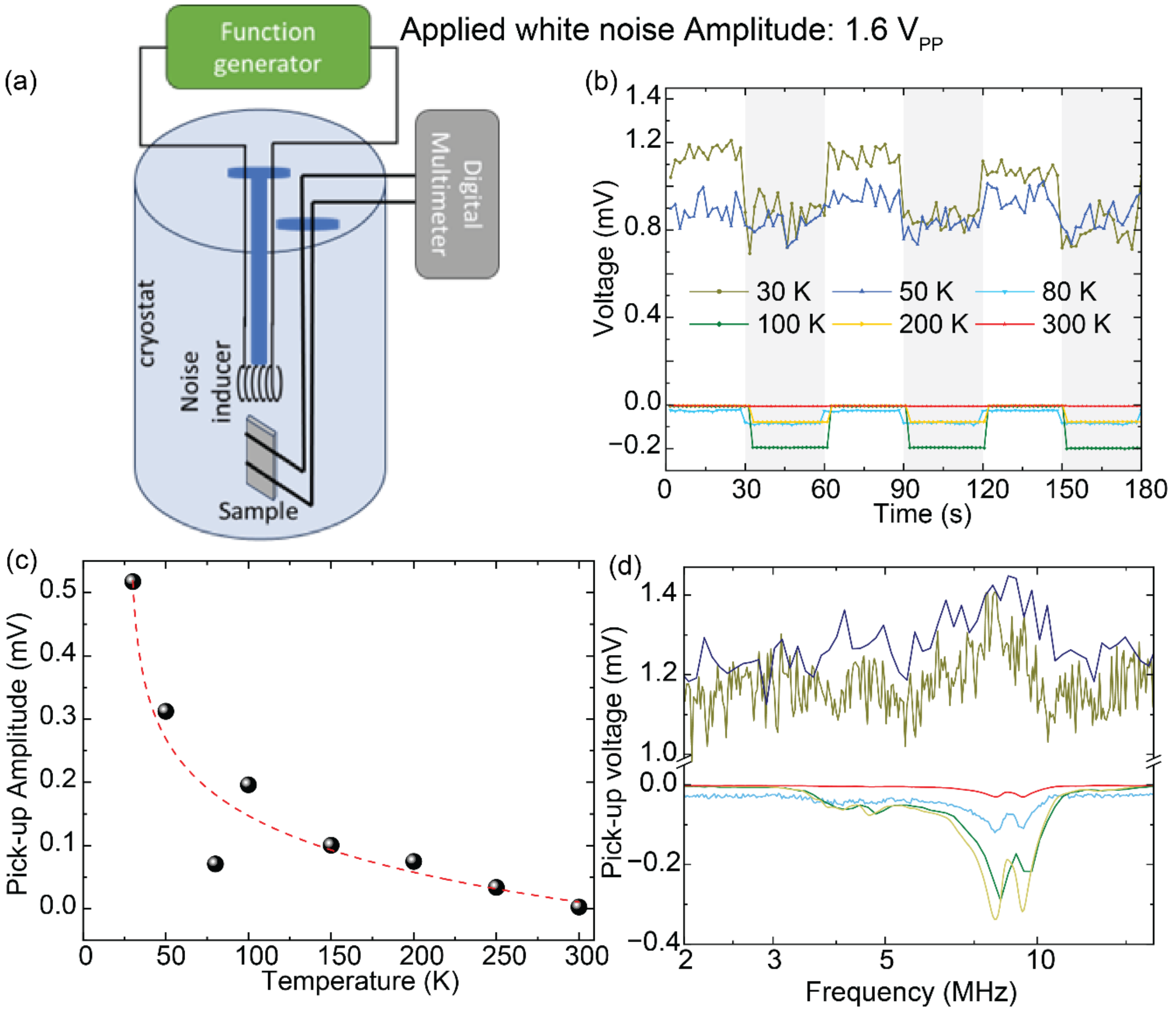

*Supplementary Figure 4: **Detection of induced noise by the sample**(a) Sketch of the setup with a coil set for inducing electromagnetic noise to the sample while its SpV signal is measured inside PPMS cryostat. (b)Time-dependence of pick-up DC voltage at 'ON' and 'OFF' state of the ac function generator as a function of sample temperature. The white and grey shaded regions are for indicating the switched off and on state of the function generator respectively. (c) Amplitude of the DC pickup voltage as a function of temperature for a constant white noise amplitude of 1.6V_{PP}. The red dotted line is a guide to the eye. (d) Frequency response of the rectified pick-up signal for a constant sine-wave amplitude of 1.6V_{PP} at different temperatures. The color code is common for Fig. 4(b) and (d).*

Supplementary Note 5: Temperature and Magnetic Field Dependence of the Second Harmonic Signal

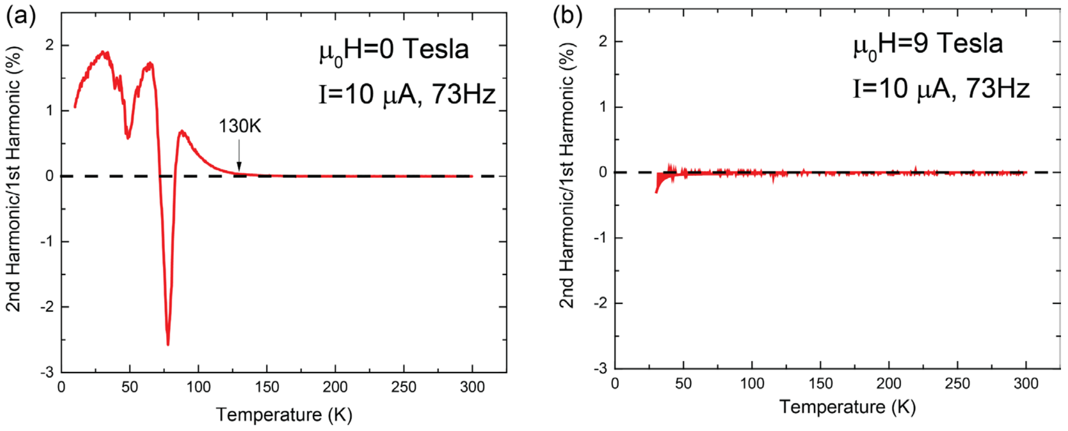

*Supplementary Figure 5: **Study of second Harmonic conversion**. Temperature and magnetic field dependence of the second harmonic component of the ac response of a NY-SL. (a) at 0 Tesla and (b) 9 Tesla.*

Supplementary Figure 5 shows the ratio of the second harmonic and the first harmonic components of the a.c. conductivity signal of an NY-SL as a function of temperature in zero magnetic field (Supplementary Figure 5 (a)) and at the highest field of 9 Tesla (Supplementary Figure 5 (b)). It was measured with a SRS SR850 lock-in amplifier as a voltage source, a reference signal generator and a current meter. A sinusoidal voltage of 0.1 V and 73 Hz was applied between the outer contacts through a series resistance of 10 kΩ while, the 1st and 2nd harmonic component of the voltage between the inner contacts of the sample, in-phase to the source were measured simultaneously.

The data in Supplementary Figure 5 confirms a close analogy with the SpV concerning the temperature dependence, with a gradually onset below about 130 K in zero magnetic field, and the magnetic field dependence, with an almost complete suppression at 9 Tesla.

This second harmonic signal is related to the formation of the rachet potential that is described in the main manuscript.

The data below ~30 K the 1st harmonic signal becomes very small and hence the ratio of the 2nd and 1st harmonic signals cannot be reliably determined. These low temperature data thus have been omitted from Supplementary Figure 5.

Supplementary Note 6: Electrical Characterization of a 10 nm thick NCSMO layer

The electrical properties of a 10-nm thick NCSMO film grown on an LSAT substrate was investigated in the same point contact geometry as shown in Figure 5 (a). Supplementary Figure 6(a) confirms the insulating ground state of NCSMO at low temperature. Meaningful measurements of the SpV signal could not be performed due to the highly insulating nature of the bare NCSMO. This leads to large noise levels in voltmeter measurements of a few hundred microVolts (Ref. Keithley Low-Level Measurement Handbook (Page. 1-4), https://download.tek.com/document/LowLevelHandbook_7Ed.pdf). The absence of a spontaneous current through an external circuit was verified by measurements with a Picoammeter (Supplementary Figure 6(b)).

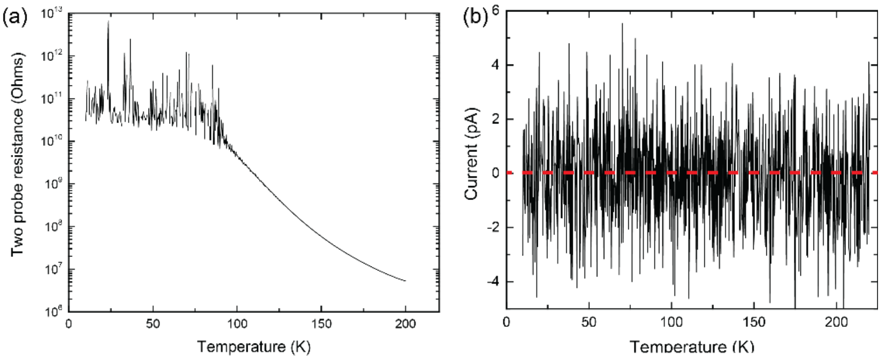

*Supplementary Figure 6: **Characterization of NCSMO layer.** (a) Resistance vs temperature, (b) current at zero external electric bias vs temperature for a single layer of NCSMO measured in point-contact geometry.*

Supplementary Note 7: Estimate of the depth of the rachet potential

Following the data[2] about polar phase properties in PrCaMnO3 with a similar position in the phase diagram of Supplementary Figure 1, we obtain the following estimates for the NSCMO layers of our heterostructures.

The reported size *l* of the polar domains amounts to about 20 nanometer and the polar moments are directed along the a-axis and give rise to head-to-head or tail-to-tail domain walls whose normal vectors are along the polar moments [2]. The estimated net polarization of $P_a \approx 40$ mC/m^2, and dielectric permittivity of the polar phase ε=35. The corresponding surface charge density σ of the domain walls amounts to about *±0.1e* per NCSMO unit cell.

$$P_a = \sigma = \frac{q}{S} = 40mC/m^2; \quad \sigma \sim 0.1\frac{e}{b \cdot c} \tag{1}$$

Here we used the NCSMO unite cell size that is doubled along the b-axis with b=7.67 Å and c = 5.42 Å. This unit cell contains four Mn ions.

The internal electric field strength *E* in the polar domain can be estimated as:

$$E = \frac{P_a}{\varepsilon_0 \varepsilon} \sim 1.3 \cdot 10^8 \frac{V}{m} \tag{2}$$

It yields a sizeable change of the electric potential *U* of about 2.6V between the opposite types of domain walls in the single polar domain:

$$U = E \cdot l = \frac{P_a}{\varepsilon_0 \varepsilon} l \sim 2.6\ V \tag{3}$$

This value defines the scale of the potential difference between the maxima and minima of the rachet potential as shown in the Fig.7 of the manuscript.

Supplementary References:

1 Tokura, Y. Critical features of colossal magnetoresistive manganites. *Rep. Prog. Phys.* **69**, 797 (2006).

2 Jooss, C. *et al.* Polaron melting and ordering as key mechanisms for colossal resistance effects in manganites. *PNAS* **104**, 13597-13602 (2007).
